# Effects of High Salinity and Water Stress on Wetland Grasses from the Spanish Mediterranean Coast

**DOI:** 10.3390/plants13141939

**Published:** 2024-07-15

**Authors:** Adrián Sapiña-Solano, Monica Boscaiu, Francisco Collado, Oscar Vicente, Mario X. Ruiz-González

**Affiliations:** 1 Institute for Conservation and Improvement of Valencian Agrodiversity (COMAV), Universitat Politècnica de València, Camino de Vera s/n, 46022 Valencia, Spain; adsaso@doctor.upv.es; 2 Mediterranean Agroforestry Institute (IAM), Universitat Politècnica de València, Camino de Vera s/n, 46022 Valencia, Spain; mobosnea@eaf.upv.es; 3 Servici Devesa-Albufera, Vivers Municipals de El Saler, CV-500, km 8.5, 46012 Valencia, Spain; fjcollado@valencia.es

**Keywords:** salt stress, water deficit, *Imperata cylindrica*, *Phragmites australis*, *Saccharum ravennae*, biotic interactions, mycorrhizae, ion content, osmolytes, photosynthetic pigments

## Abstract

The impacts of climate change are reaching unprecedented levels, heightening the risk of species loss and ecosystem service degradation. Wetlands, highly threatened ecosystems, serve vital ecological functions by capturing carbon, filtering water, and harbouring diverse wildlife. Coastal wetlands encounter many challenges, such as increased drought periods and escalating salinity levels, severely impacting plant biodiversity. Assessing how plants respond to various environmental stress factors is imperative for devising successful conservation strategies. In the present study, we examined three representative grass species found in various habitats within the Albufera Natural Park, close to the city of Valencia on the Spanish Mediterranean coast: *Imperata cylindrica*, *Phragmites australis*, and *Saccharum ravennae*. High salinity and water stress conditions were induced by subjecting the plants to irrigation with solutions containing 200, 400, 600, and 800 mM NaCl or withholding irrigation altogether to mimic coastal flooding and drought scenarios. The treatments were maintained until noticeable wilting of the plants occurred, at which point a range of stress biomarkers were determined, including photosynthetic pigments, ions, osmolytes, oxidative stress markers, and antioxidant metabolites, as well as antioxidant enzyme activities. *Saccharum ravennae* displayed the highest sensitivity to salt stress, whereas *I. cylindrica* appeared to be the most tolerant. The primary salinity tolerance mechanism observed in *I. cylindrica* and *P. australis* was a blockage of ion transport from the root zone to the aerial part, together with the salt-induced accumulation of proline and soluble sugars to high concentrations in the former. No significant effects of the water deficit treatment on the growth or biochemical parameters were observed for any of the analysed species. These findings offer valuable information for the effective management and conservation of coastal wetlands facing the challenges posed by climate change.

## 1. Introduction

One of the threats associated with climate change is sea level rise, with the consequent risk of inundating critical ecosystems such as coastal wetlands and dune systems [1,2,3]. Coastal wetlands provide essential ecosystem services [4,5,6]; however, they are heavily threatened by pollution [7,8], invasive species [9], the overexploitation of biological resources [10], human activities such as urbanisation or agriculture [11], and, especially, the effects of climate change [12,13].

Amongst the latter effects, drought and soil salinisation are the abiotic factors that have a major impact on plants and soil organisms [14]. Mediterranean wetlands are both biodiversity hotspots and fragile environments [15]. These areas harbour numerous endemic taxa and provide refuge for transient species [16]. However, due to the changing environmental conditions, accelerated by anthropogenic activity and climate change, new or emerging abiotic stress conditions may drastically affect those endemic species. The most likely abiotic stresses in these ecosystems are water deficit and salinisation [1,2]. In this context, the species most sensitive to salt stress could become locally extinct, favouring the expansion of more tolerant species, whether native or allochthonous, which could destabilise the ecosystem balance [17].

The Albufera Natural Park (Valencia, Spain) is an important and protected wetland area on the Mediterranean coast, which has been included in the Ramsar Convention (https://www.ramsar.org/, accessed on 20 December 2023) and designated as a Special Protection Area (Natura 2000, ES0000471). The natural park consists of different ecosystems: the coastal band, primary and secondary sand dunes, salt marshes, wetlands, pine dune woodlands, and the freshwater Albufera Lake. The natural park hosts a characteristic halophytic flora and rare and endemic species of high ecological value. Although many species inhabiting this ecosystem are halotolerant or halophytic, their degree of tolerance is variable, and they are distributed along salinity gradients according to their relative salt tolerance [18,19]. However, soil salinity is not the only stress factor for the species present in these ecosystems as they must also tolerate intense droughts that may extend beyond the Mediterranean summers [20].

A prevalent family found at the Albufera Natural Park is the Poaceae, with the species adapted to all the ecosystems, from *Elymus farctus* L. in the first line of dunes to *Phragmites australis* (Cav.) Trin. ex Steud. in the wetlands, as well as invasive species such as *Arundo donax* Forssk. or *Spartina patens* Muhl. Thus, the park is inhabited by 75 species of Poaceae [21] that fulfil vital ecological functions, such as dune fixation, water purification, carbon sequestration, or providing food and shelter to wildlife. These species resist adverse conditions and establish symbiotic interactions with mycorrhizal fungi, which are present on the roots of most terrestrial plants [22]. This symbiosis is a valuable biological tool, especially in saline environments, enhancing their resistance to abiotic stress and promoting plant growth [23,24,25,26].

*Imperata cylindrica* (L.) Raeusch., *Phragmites australis*, and *Saccharum ravennae* (L.) L. are three monocotyledonous grass species that, in addition to establishing the symbiotic interactions mentioned above, display tolerance to salt and water stress [26,27,28]. The characterisation of the strength of these tolerance mechanisms might provide valuable information on the resilience of these ecosystems to drought and high salinity stress.

*Imperata cylindrica*, cogon grass, spear grass, or red baron, is native to Africa, southern Europe, and Southeast Asia; it is widely distributed in tropical and subtropical regions, including Pakistan, the Mediterranean region, the Middle East, South America, and the United States [29,30]. Due to its survival strategies, *I. cylindrica* has strong invasiveness potential: it is an aggressive, coarse, and rhizomatous perennial herb that produces large quantities of anemochore seeds and tolerates poor soils, nutrient deficiency, and both drought and salinity, with specific morphological and physiological adaptations to high-salinity conditions, and exhibits fire adaptability and genetic plasticity [27,31,32]. Moreover, it can modify soil microbial communities and root colonisation [33]. In addition, due to its effects on the commercial forestry, agriculture, and fire regimes in the natural ecosystems in the southeastern United States and tropical and subtropical areas of Asia and Africa, this species is included within the ten worst weeds in the world, being considered a pest in over 73 countries [30,31,34,35,36,37]. It can, however, provide different services, such as soil stabilisation, animal fodder and grazing areas, and thatch or paper production [30].

The common reed, *Phragmites australis*, is widespread in the temperate regions of the world and colonises habitats ranging from marshes and wetlands to riverbanks and lakes [38]. There are several ecotypes of this species that have developed resistance to different types of environmental stress, such as drought, salinity, or low temperatures [38,39,40,41]. Therefore, *P. australis* plays an essential role in ecosystems such as coastal wetlands; it contributes to soil building and stabilisation and promotes the formation of persistent vegetation in urban and industrial areas, where other plant species struggle to thrive, as well as sequestering nutrients, heavy metals, and carbon [42]. Furthermore, due to its remarkable ability to withstand harsh environmental conditions, it has become a target in the United States due to its invasiveness [43,44].

*Saccharum ravennae* (≡ *Erianthus ravennae* (L.) P. Beauv.) is a grass native to Eurasia and North Africa [45]. It has been introduced in Australia, Japan, and the USA, where it is commonly used in ornamental plantings and has escaped cultivation, becoming naturalised in many areas [46,47]. Its adaptability is reflected in its ability to withstand adverse conditions, making the species of interest not only for ornamental purposes but also for erosion control, genetic research, and bioenergy production [45,48,49]. *Saccharum* spp. provide several uses, such as timber, fodder, medicine, pulp, soil conservation, and bioethanol production. Moreover, wild *Saccharum* spp. represent a potential base for the genetic improvement of sugar cane and have a strong potential in ecological restoration and bioremediation [50].

Mediterranean coastal systems are exposed to several projected, moderate to high, climate risks, such as erosion, flooding due to sea level rise, warming, heat waves, and drought [51]. Sea level rise can result in saltwater intrusion and soil salinisation, a problem already happening in Spain. Plants respond to salt stress, which can result in detrimental effects such as osmotic stress and ionic toxicity by activating physiological, biochemical, and molecular mechanisms regardless of their inherent salt tolerance. High salt concentrations also interfere with mineral nutrition and increase the reactive oxygen species (ROS) levels [52]. ROS accumulation damages membrane permeability and leads to protein inactivation, DNA mutations, and cell death [53]. In response, plants activate antioxidant systems, such as antioxidant enzymes and metabolites, and accumulate specific osmolytes to counteract the oxidative stress [54]. In addition, high concentrations of Na^+^ and Cl^−^ ions, resulting from salt stress, are toxic and lead to the inhibition of enzymatic activities, thus affecting the cellular structures and functions [55]. To avoid the damage caused by salt stress, monocotyledonous halophytes block the ion transport from the root zone to the aerial parts of the plant [56,57].

In this work, we have investigated the responses of *Imperata cylindrica*, *Phragmites australis*, and *Saccharum ravennae* exposed to four salt treatments (200, 400, 600, and 800 mM NaCl) and to water stress, mimicking possible natural scenarios in the context of climate change. These species are exposed to risk due to their proximity to the sea. Within the Albufera Natural Park, *I. cylindrica* thrives in areas of marshes with moderate levels of salinity; *Phragmites australis*, on the other hand, is found in marshes with a wide range of salinities, from freshwater to high salt concentrations, whereas *Saccharum ravennae* is usually found in dry riverbeds, riverine sandbanks, moist coastal sands, the margins of watercourses, or depressed areas with a high water table [58,59]. Thus, our working hypothesis states that the ecology of the species will reflect their ability to cope with stress. Studying plant tolerance to abiotic stress in wetlands is urgently needed as these ecosystems face increasing threats from climate change, pollution, and habitat degradation, which can significantly impact their biodiversity and functionality. To evaluate the possible mechanisms involved in the response of these plants to high salt and water stresses, we have analysed different biochemical stress markers and enzymatic and non-enzymatic components of the response to oxidative stress. In addition, we have explored the presence of mycorrhizae in the three species because of their important role in enhancing wetland plants’ responses to abiotic stress [60]. Our work will provide valuable information on the resilience of these species against high salt and water stress events and will contribute to the design and implementation of efficient management, conservation, and regeneration programmes for preserving these threatened habitats and maintaining their ecological services in a rapidly changing scenario.

## 2. Results

### 2.1. Effect of High Salinity and Water Stress Treatments on Plant Biomass

The first distinct effect that the application of the highest-salinity treatment (800 mM NaCl) had on the plants was the time it took for them to show signs of wilting. This strongly relates to the accumulation of salt in the pots during the treatments, reflected in the progressive increase in the substrate electrical conductivity (Table 1). The *Imperata cylindrica* plants resisted for 14 days, *P. australis* for 11 days, and *S. ravennae* was the most sensitive to salt stress, exhibiting drastic wilting symptoms after seven days. The high salt and water stress treatments had different significant overall effects on the biomass of the selected plant species except for the root water contents (Table 2). The treatments had significant general effects on the leaf and root fresh weight, the production of new leaves and stems, and the root/shoot ratio in *I. cylindrica*, on the number of leaves and stems produced in *P. australis*, and on the leaf fresh weight, leaves and stems produced, leaf water content, and root/shoot ratio in *S. ravennae* (Table 2).

**Figure 1:**
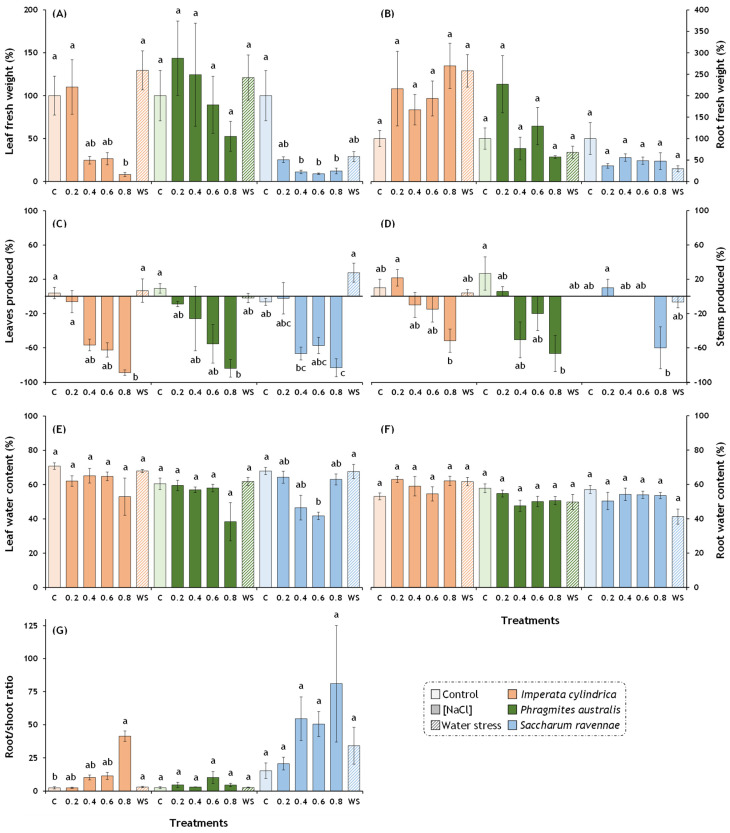
Effects of salt and water stress treatments on plant biomass. Thirty plants of each species (five plants per treatment) were exposed to four salt concentrations (0.2, 0.4, 0.6, and 0.8 M NaCl), water stress (WS, withholding of irrigation) on pots containing fresh soil from the Albufera Natural Park, and control plants (C). Control plants were watered twice weekly with 2 L of tap water (EC = 1.051 mS/cm). Treatment lengths were 14 days for *I. cylindrica*, 11 days for *P. australis*, and seven days for *S. ravennae*. (**A**) Leaf fresh weight; (**B**) root fresh weight; (**C**) leaves produced or lost during the treatment; (**D**) stems produced or lost during the treatment; (**E**) leaf water content; (**F**) root water content; (**G**) root/stem ratio. Bars represent mean ± SE. Letters denote post hoc significant pairwise differences between treatments corrected after Bonferroni. Statistical analyses were performed independently for each species.

Salt stress had negative effects on the leaf fresh weight, the production of leaves and stems, and increased the root/shoot ratio in *I. cylindrica* (Figure 1A,C,D,G), whereas, in *P. australis*, salt stress only reduced the production of leaves and stems (Figure 1C,D). In *S. ravennae*, salt stress strongly negatively affected the leaf fresh weight, leaf and stem production, and leaf water content (Figure 1A,C–E). The water stress treatment, however, did not produce significant differences in any biomass marker compared to the control plants.

### 2.2. Photosynthetic Pigments

**Figure 2:**
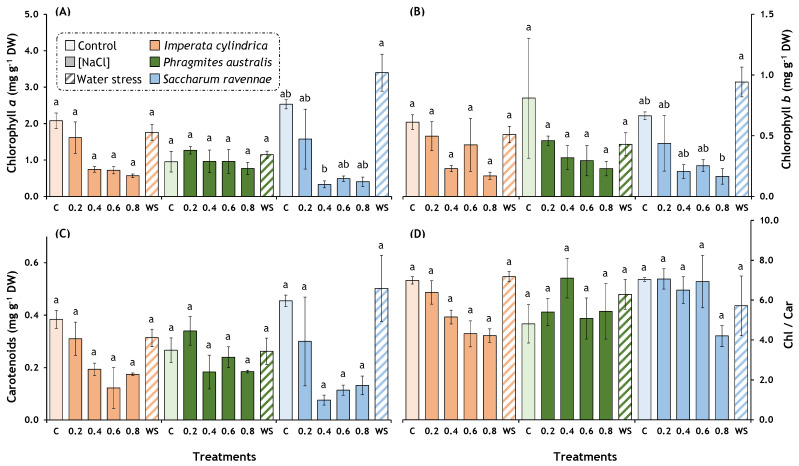
Effects of salt and water stress treatment on photosynthetic pigments of *Imperata cylindrica*, *Phragmites australis*, and *Saccharum ravennae*. Thirty plants of each species (five plants per treatment) were exposed to four salt concentrations (0.2, 0.4, 0.6, and 0.8 M NaCl), water stress (WS, withholding of irrigation) on pots containing fresh soil from the Albufera Natural Park, and control plants (C). Control plants were watered twice weekly with 2 L of tap water (EC = 1.051 mS/cm). Treatment lengths were 14 days for *I. cylindrica*, 11 days for *P. australis*, and 7 days for *S. ravennae*. (**A**) Chlorophyll *a*; (**B**) chlorophyll *b*; (**C**) carotenoids; (**D**) total chlorophylls/carotenoids ratio. Bars represent mean ± SE. Letters denote post hoc significant pairwise differences between treatments corrected after Bonferroni. Statistical analyses were performed independently for each species.

Significant overall effects of the treatments were found for *I. cylindrica* in all the photosynthetic pigment variables (chlorophylls *a* and *b*, carotenoids, and the chlorophyll/carotenoid ratio) and for *S. ravennae* in chlorophylls *a* and *b* (Table 2). Salt stress significantly decreased the concentration of chlorophylls *a* and *b* in *S. ravennae*, reducing the Chl *a* content up to 84% and the Chl *b* content up to 75% (Figure 2A,B). A similar pattern (non-significant) was observed in *I. cylindrica* for Chl *a*, carotenoids, and the chlorophyll/carotenoids ratio (Figure 2A,C,D), as well as in *P. australis* for Chl *a* and *b* (Figure 2A,B).

### 2.3. Ion Contents

**Figure 3:**
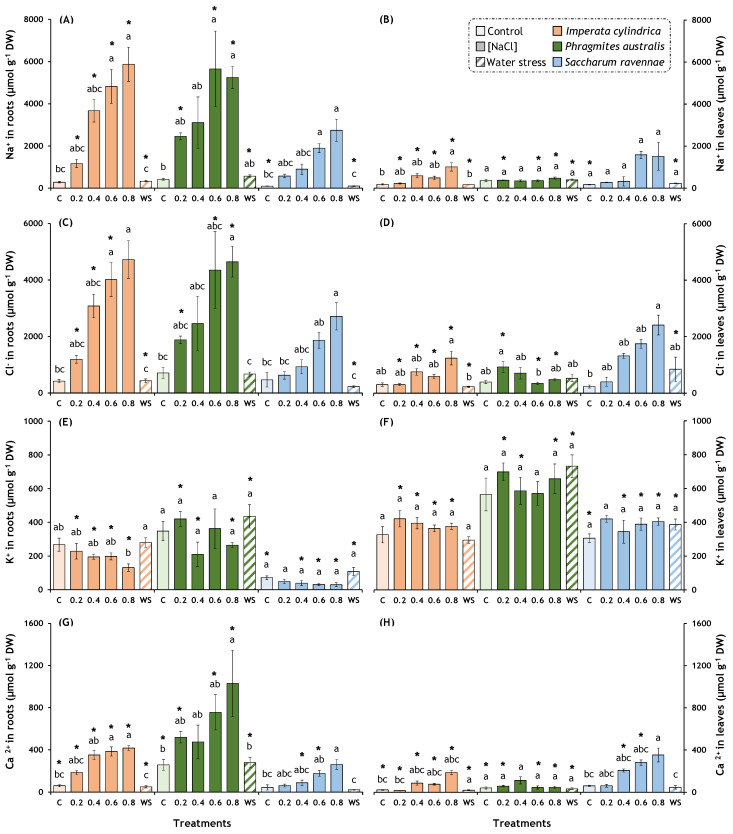
Effects of salt and water stress treatments on ions contents in *Imperata cylindrica*, *Phragmites australis*, and *Saccharum ravennae* roots and leaves. Thirty plants of each species (five plants per treatment) were exposed to four salt concentrations (0.2, 0.4, 0.6, and 0.8 M NaCl), water stress (WS, withholding of irrigation) on pots containing fresh soil from the Albufera Natural Park, and control plants (C). Control plants were watered twice weekly with 2 L of tap water (EC = 1.051 mS/cm). Treatment lengths were 14 days for *I. cylindrica*, 11 days for *P. australis*, and 7 days for *S. ravennae*. (**A**,**B**) Na^+^ ions in roots and leaves; (**C**,**D**) Cl^−^ ions in roots and leaves; (**E**,**F**); K^+^ ions in roots and leaves; (**G**,**H**) Ca^2+^ ions in roots and leaves. Bars represent mean ± SE. Letters denote post hoc significant pairwise differences between treatments corrected after Bonferroni. Asterisks indicate significant differences between roots and leaves for the same treatment and species. Statistical analyses were performed independently for each species.

The high salt and water stress treatments produced significant overall effects on the ion contents in the roots and leaves (Table 2). In *I. cylindrica*, the treatments had overall effects on the Na^+^ concentration in the roots and leaves, the Cl^−^ concentration in the roots and leaves, the K^+^ concentration in the roots, and the Ca^2+^ concentration in the roots and leaves (Table 2). Similarly, in *P. australis*, the treatment affected the Na^+^ concentration in the roots, the Cl^−^ concentration in the roots and leaves, and the Ca^2+^ concentration in the roots. Finally, in *S. ravennae*, the treatment affected the Na^+^ concentration in the roots, the Cl^−^ concentration in the roots and leaves, the K^+^ concentration in the roots, and the Ca^2+^ concentration in the roots and leaves (Table 2). In *I. cylindrica*, salt stress increased the concentration of Na^+^ up to 20-fold in the roots and 5.5-fold in the leaves; Cl^−^ increased up to 11.2-fold in the roots and 4.1-fold in the leaves; and Ca^2+^ increased up to 6.8-fold in the roots and 8.4-fold in the leaves but decreased the concentration of K^+^ up to 50% in the roots (Figure 3A–E,G,H). Similarly, in *P. australis*, salt stress increased the concentration of Na^+^ up to 13.6-fold in the roots and up to 1.3-fold in the leaves; Cl^−^ increased up to 6.6-fold in the roots and 1.8-fold in the leaves; and Ca^2+^ increased up to 4-fold in the roots and 2.8-fold in the leaves (Figure 3A,C,D,G). Likewise, in *S. ravennae*, salt stress increased the concentration of Na^+^ up to 28.5-fold in the roots and 9.3-fold in the leaves; Cl^−^ and Ca^2+^ increased up to 5.8-fold in the roots and up to 10.2-fold in the leaves, and up to 6-fold in the roots and up to 5.8-fold in the leaves, respectively (Figure 3A,C,D,G,H). Water stress did not produce significant differences compared to the control plants.

The concentration of Na^+^ was significantly higher in the roots than in the leaves for the salt and water stress treatments in *I. cylindrica*. The same trend was observed in *P. australis* except for the 400 mM NaCl treatment (Figure 3A,B). Similar findings were detected for Cl^−^ and Ca^2+^ (Figure 3C,D,G,H), although the *S. ravennae* plants treated with 400 and 600 mM NaCl showed higher concentrations of Ca^2+^ in the leaves than in the roots. The concentration of K^+^ was significantly lower in the roots than in the leaves for the three species (Figure 3E,F).

### 2.4. Osmolytes

**Figure 4:**
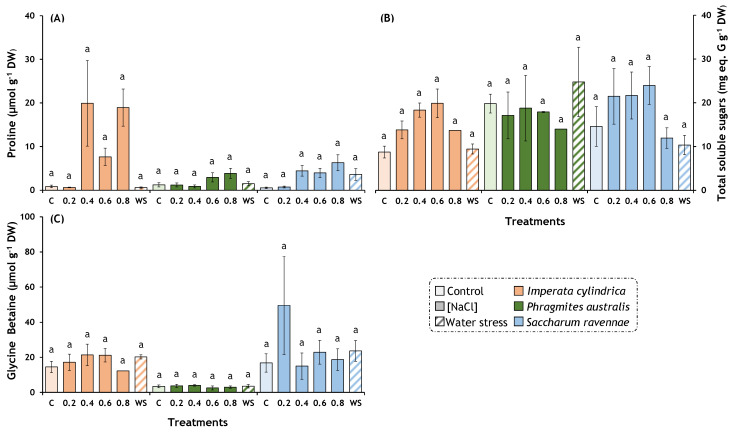
Effects of salt and water stress treatments on osmolyte contents in *Imperata cylindrica*, *Phragmites australis*, and *Saccharum ravennae*. Thirty plants of each species (five plants per treatment) were exposed to four salt concentrations (0.2, 0.4, 0.6, and 0.8 M NaCl), water stress (WS, withholding of irrigation) on pots containing fresh soil from Albufera Natural Park, and control plants (C). Control plants were watered twice weekly with 2 L of tap water (EC = 1.051 mS/cm). Treatment lengths were 14 days for *I. cylindrica*, 11 days for *P. australis*, and 7 days for *S. ravennae*. (**A**) Proline; (**B**) total soluble sugars; (**C**) glycine betaine. Bars represent mean ± SE. Letters denote post hoc significant pairwise differences between treatments corrected after Bonferroni. Statistical analyses were performed independently for each species.

The high salt and water stress treatments had significant overall effects on the proline and total soluble sugar concentrations but not on glycine betaine (Table 2). In *I. cylindrica*, the treatments had overall effects on the proline content and total sugar content, whereas, in *S. ravennae*, the treatment only affected the proline content. No significant post hoc pairwise comparisons were detected within each species for the treatments. However, a positive trend in the proline content, total sugar content, and glycine betaine content (non-significant) was observed with increasing salt concentrations in *I. cylindrica*. The same trend (non-significant) was observed for the proline content with increasing salt concentrations in *S. ravennae* and *P. australis* (Figure 4A–C).

### 2.5. Oxidative Stress Biomarkers and Antioxidant Compounds

The high salt and water stress treatments only produced significant overall effects on the malondialdehyde content, total phenolic compounds, and total flavonoids in *I. cylindrica* and on the glutathione reductase (GR) activity in *S. ravennae* (Table 2). However, the post hoc pairwise analyses were not statistically significant (Table 3).

### 2.6. Quantification of Mycorrhiza

**Figure 5:**
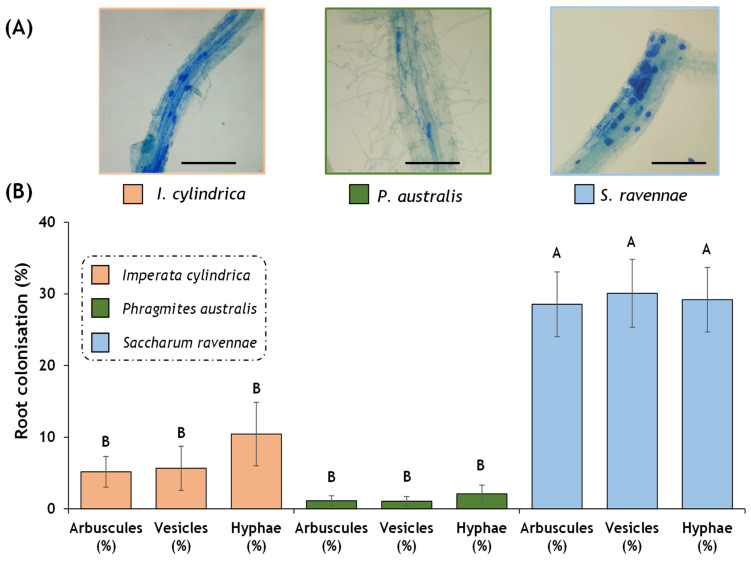
Percentage of mycorrhiza colonisation (arbuscules, vesicles, and hyphae) in *Imperata cylindrica*, *Phragmites australis*, and *Saccharum ravennae* roots. (**A**) Example of mycorrhization status in *I. cylindrica*, *P. australis*, and *S. ravennae*. Roots were stained with acidified Shaeffer blue ink. The scale corresponds to 500 µm. (**B**) Percentage of root colonisation in *I. cylindrica*, *P. australis*, and *S. ravennae* roots. Bars represent mean ± SE. Capital letters denote post hoc significant pairwise differences between species for each mycorrhizae structure corrected after Bonferroni.

*Saccharum ravennae* showed the highest percentage of mycorrhizal colonisation compared to *I. cylindrica* and *P. australis* (Figure 5B). Thus, the presence of arbuscules, vesicles, and hyphae on the roots was higher in *S. ravennae* compared to *I. cylindrica* and *S. ravennae* (all *p*-values < 0.001).

### 2.7. Principal Component and Correlation Analyses

**Figure 6:**
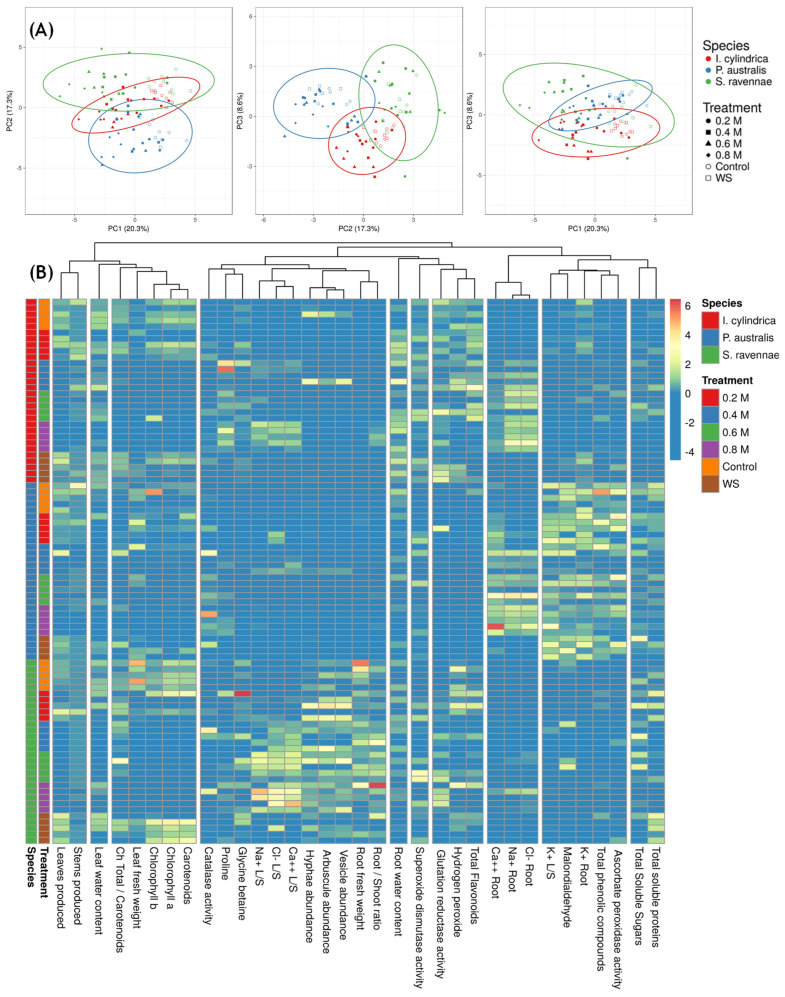
PCA analysis and heatmap for species and treatment. (**A**) Contribution of the three main components to data variance by treatment. (**B**) Manhattan distance and complete linkage heatmap of treatment effects on *I. cylindrica*, *P. australis*, and *S. ravennae*. The seven groups of variables explained 67% of the variance.

The PCA and the heat map (Figure 6) show the impact of the salt and water stress treatments on *I. cylindrica*, *P. australis*, and *S. ravennae* with respect to the plant biomass and stress response biomarkers (Figure 6). Seven principal components accounted for 67% of the total variance. The PC2 nicely separates the three species, although *P. australis* and *S. ravennae* clearly separate into two distinct groups (Figure 6A). PC1 separates the control and water-stressed from the salt-stressed plants (Figure 6A), as shown in the above results. The heatmap enabled detecting some interactions (Figure 6B): the pigment concentrations were higher in the control and water stress than in the salt-treated plants. *Saccharum ravennae* was the species that accumulated the most Na^+^, Cl^−^, and Ca^2+^ in the leaves. *Phragmites australis* showed a group of variables with values higher than those in the other species: K^+^ in the roots and leaves, malondialdehyde, ascorbate peroxidase activity, and total phenolic compounds (Figure 6B).

**Figure 7:**
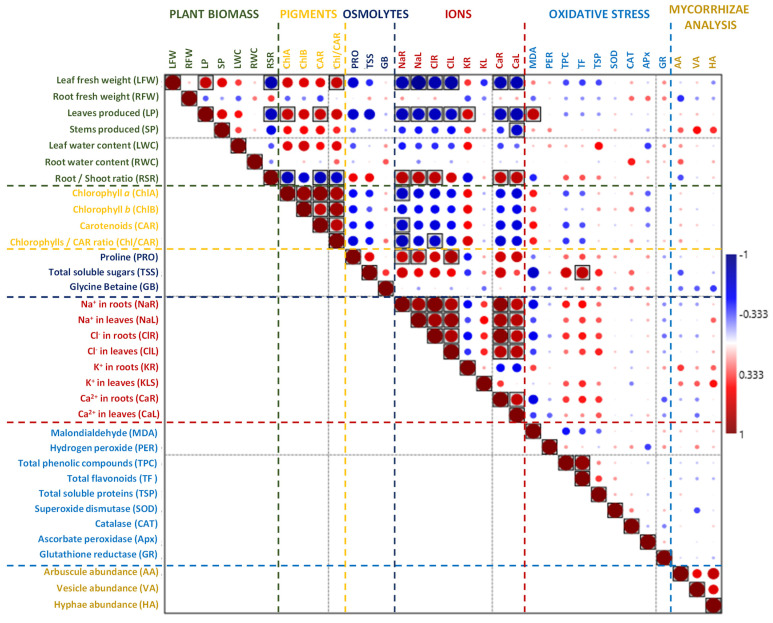
Spearman’s rho correlation between biomass and stress markers in *I. cylindrica*. Red fillings denote positive and blue fillings negative correlations. Significant correlations after Bonferroni correction are shown in boxes (*p*-value < 0.05).

In *I. cylindrica*, the partial correlation analysis detected a positive interaction between the leaf fresh weight and the chlorophyll/carotenoid ratio. In addition, chlorophyll *a* and *b*, root K^+^, and MDA positively interacted with the number of leaves produced. The Na^+^ content in the roots and leaves, the Ca^2+^ content in the roots and leaves, and the Cl^−^ content in the roots demonstrated positive interactions with the root/shoot ratio. On the other hand, there was a negative interaction between chlorophyll *a*, carotenoids, and chlorophyll/carotenoids with the root/shoot ratio. The Na^+^, Cl^−^, and Ca^2+^ contents in the roots and leaves also demonstrated negative interactions with the leaf fresh weight and the number of leaves produced (Figure 7).

**Figure 8:**
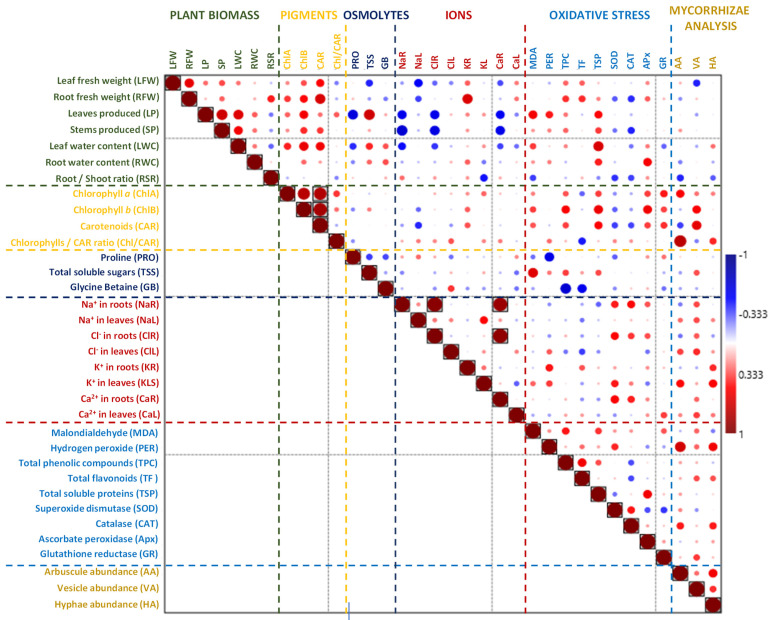
Spearman’s rho correlation between biomass and stress markers in *P. australis*. Red fillings denote positive and blue fillings negative correlations. Significant correlations after Bonferroni correction are shown in boxes (*p*-value < 0.05).

**Figure 9:**
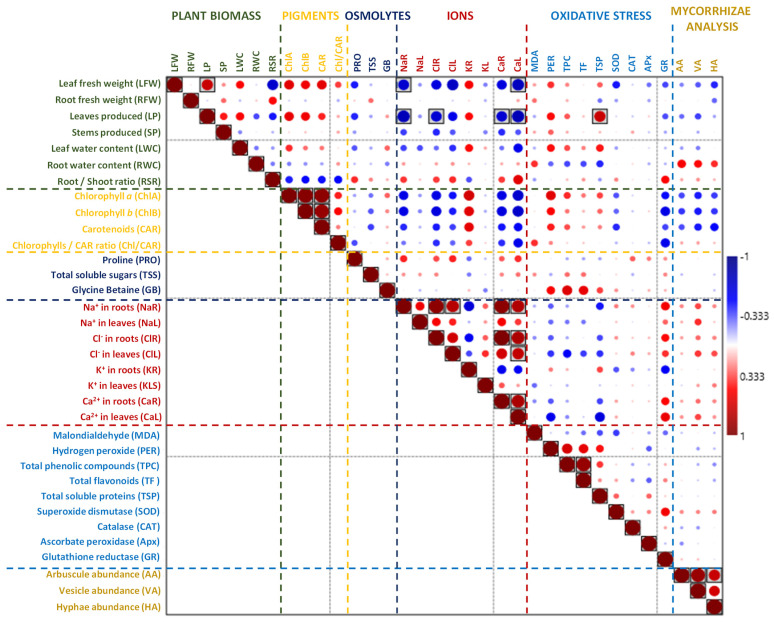
Spearman’s rho correlation between biomass and stress markers in *S. ravennae*. Red fillings denote positive and blue fillings negative correlations. Significant correlations after Bonferroni correction are shown in boxes (*p*-value < 0.05).

In *P. australis*, no positive or negative correlation was detected between the biomass parameters and the other variables analysed (Figure 8). In *S. ravennae*, the partial correlation analysis detected a positive interaction between the amount of soluble protein and the number of leaves produced. The analysis detected a negative correlation between the Na^+^ content in the roots and the Ca^2+^ content in the leaves and the leaf fresh weight, and there was also a negative interaction between the Na^+^ content in the roots, the Cl^−^ content in the roots, and the Ca^2+^ content in the roots and leaves and the number of leaves produced (Figure 9).

Overall, the control, mild salt stress, and water stress conditions had little or no effect on the plants, highlighting that high salt stress is the main challenge for these species. We found large variability for some characteristics when plants of different species were exposed to salt stress above 400 mM NaCl, which may be due to the genetic differences of the individual plants, in addition to the low sample size.

## 3. Discussion

Our results confirm the higher resistance of *I. cylindrica* and *P. australis* to salinity, compared to *S. ravennae*, which seems to be mainly based on constitutive defence mechanisms such as blocking ion transport from the roots to the aerial part of the plants. In addition, the possible activation of K^+^ transport into the leaves under high-salinity conditions could contribute to their increased tolerance.

*Imperata cylindrica* showed other tolerance mechanisms, such as the accumulation of osmolytes (proline and soluble sugars) to maintain osmotic balance. It also increased the accumulation of antioxidant metabolites (total phenolic compounds and total flavonoids), which probably helped to counteract the deleterious effects of the salt-induced oxidative stress. Therefore, *I. cylindrica* might be the most suitable species for slowing down the erosion of soils exposed to salinisation.

Despite activating antioxidant enzymes, such as GR, and having a higher degree of mycorrhizal colonisation, *S. ravennae* was the least salt-tolerant species as it was the first to show wilt symptoms.

When facing increased salt concentrations, *I. cylindrica*, *P. australis*, and *S. ravennae* reduced their fresh weights and lost leaves and stems. This response is due to a trade-off between allocating plant resources for biomass accumulation and activating the stress tolerance mechanisms [52,61]. Plant biomass production depends on the accumulation of carbon products through photosynthesis. However, the presence of high salinity levels can negatively affect photosynthesis [27,61,62,63,64], leading to a reduction in plant biomass. There is evidence of the effect of increased salt concentration on plant biomass and a reduction in the number of leaves and stems in these species [27,28,40]. In addition, under low salt and water stress, *S. ravennae* exhibits greater variability in leaf wilting compared to control (healthy) conditions or high salt concentrations (wilted).

High salinity and a water deficit in the soil generate osmotic stress in the plants, leading to leaf dehydration [52]. However, *I. cylindrica* and *P. australis* seem to be tolerant to this osmotic effect because no significant water loss was detected in the presence of high NaCl concentrations, or even after a period without irrigation. This ability to retain water suggests efficient adaptive mechanisms in these species to counteract the osmotic stress associated with salinity or a lack of water [28,65]. However, *S. ravennae* experienced a substantial decrease in the aerial water content, indicating greater sensitivity to the osmotic stress conditions and a less efficient response to regulate the water balance under high salinity.

Our experiments detected a decrease in the chlorophyll *a* and *b* levels with increasing salt concentrations in all three species. This decrease in the chlorophyll content is caused by the inhibition of the enzymes involved in chlorophyll biosynthesis, such as Rubisco and PEP carboxylase, and the activation of chlorophyllase, which breaks down chlorophyll [66,67]. The reduction in the chlorophyll *a* and *b* content was more noticeable in *S. ravennae* than in *I. cylindrica* and *P. australis*, which appear to have more efficient mechanisms to preserve the chlorophyll integrity, crucial for photosynthesis, under salt stress conditions. The more pronounced response in *S. ravennae* suggests a greater susceptibility of this species to salinity, manifested in a more marked reduction in the photosynthetic pigments. Research by Hameed et al. [64] supports our findings by showing a decrease in chlorophylls *a* and *b* and carotenoids due to an increasing salt concentration in *I. cylindrica*. Furthermore, similar results were obtained by Gorai et al. [27] in *P. australis*.

*Imperata cylindrica* and *P. australis* exhibited a blockage of ion transport from the root zone to the aerial part, suggesting an effective strategy to mitigate the adverse effects of salt stress. Similar results have been observed in *P. australis* [26,27,28,41]. This was expected because a common response to salt stress in many plant species, contributing to tolerance, is based on blocking ion transport. Indeed, glycophytes and monocotyledonous halophytes cope with high salinity mainly by limiting the uptake and transport of Na^+^ and Cl^−^ to the leaves [68,69,70]. However, this blockade of ion transport was not observed in *S. ravennae*, which could indicate a less efficient response to exposure to high salinity. Our experiments also detected a significant decrease in the K^+^ levels in the roots of *I. cylindrica* as the Na^+^ concentrations increased. However, the K^+^ levels in the aerial part remained high in all three species. This result suggests the activation of K^+^ transport to the leaves, which could partly compensate for Na^+^ accumulation and, thus, contribute to salt tolerance.

In general, the accumulation of Na^+^ is accompanied by a reduction in the K^+^ levels in plants as both cations compete for the same binding sites on proteins, including membrane channels and K^+^ transporters [68,69,70]. Given the fundamental role of K^+^ in photosynthesis, osmoregulation, turgor generation, membrane potential regulation, and essential cellular functions such as protein synthesis and enzyme activity, it seems clear that the adverse effects of salt stress are due, at least in part, to decreased cytosolic K^+^ concentrations, especially in photosynthetic tissues [71]. Therefore, maintaining a relatively high cytosolic K^+^ level (mainly in leaf cells) is another fundamental tolerance mechanism described in some halophytes [72].

*Imperata cylindrica* displayed a significant increase in the proline levels and a trend to increase the concentrations of the total soluble sugars and glycine betaine in response to the rising salt concentrations. These findings are consistent with those of Hameed et al. [64]. The accumulation of proline, soluble sugars, and glycine betaine in *I. cylindrica* suggests the activation of inherent stress response mechanisms in this species. When these molecules accumulate to a sufficiently high level, they help in cellular osmotic adjustments, act as ROS scavengers, and influence gene expression regulation [73,74,75], ultimately favouring stress tolerance [76,77]. Although a significant increase in the concentrations of proline and soluble sugars in *P. australis* was not observed in the present study, there is evidence of an increase in these molecules under salt stress conditions [27,28].

Only *I. cylindrica* showed an increase in the total phenol and flavonoid contents with increasing salt concentrations. The accumulation of these compounds may reduce the oxidative damage caused by abiotic stress [78,79]. In addition, flavonoids seem to constitute a secondary ROS scavenging system, activated when plants face severe stress conditions and the primary antioxidant defence systems (i.e., antioxidant enzymes) are weakened [80,81,82,83]. These results support the idea that salinity-tolerant species, such as *I. cylindrica*, use efficient mechanisms, such as accumulating potent non-enzymatic antioxidants, in response to salt stress.

Regarding the four antioxidant enzymes tested (SOD, CAT, AP, and GR), a significant overall increase in specific activity was only observed in *S. ravennae* for glutathione reductase activity. The GR enzyme plays a crucial role in the recovery and maintenance of cellular redox balance in stressed plants by reducing oxidised glutathione (GSSG) to GSH using NADPH as a cofactor [84]. However, the GR activation appears to be insufficient to counteract or limit the adverse effects of the salinity-induced oxidative stress in *S. ravennae*, given the higher sensitivity of this species.

All three species were naturally colonised by mycorrhizal fungi. Such symbiotic associations benefit plants because, amongst other reasons, they facilitate the uptake of essential nutrients. Mycorrhizal fungi have also been associated with enhancing stress tolerance in plants by strengthening the enzymatic and non-enzymatic antioxidant defence systems [85,86], affecting lipid peroxidation [87], and phytohormone synthesis [88].

*Saccharum ravennae* was observed to have a higher percentage of arbuscules, vesicles, and hyphae compared to *I. cylindrica* and *P. australis*. As discussed above, the mycorrhizal formation can improve the plants’ ability to take up nutrients. Given the higher susceptibility of this species, *S. ravennae* may have more effective strategies for using mycorrhizal symbiosis to overcome the nutritional limitations in a saline environment.

Rising sea levels, as a consequence of climate change, could cause a drastic increase in soil salinity and, therefore, changes in biodiversity, ecology, and ecosystem balance in areas close to the coast. Species that do not possess efficient salt stress tolerance mechanisms, such as *S. ravennae*, might be displaced or driven to local extinction, along with many other endemic species that provide essential ecosystem services. Then, the opportunity arises for tolerant species to prosper and even facilitate the establishment of invasive species. The main consequence is the drastic alteration of the population structure of native species. Thus, careful observation of sea flooding events in coastal areas where *S. ravennae* prospers should become an activity for monitoring the natural park. Another severe consequence is when the local salt-tolerant species, such as *I. cylindrica* or *P. australis*, cannot compete with the invader species, e.g., *Spartina patens*. The latter can reduce the presence and coverage of the native species, altering the original vegetation structure. Moreover, it has been reported that *S. patens* can transform the original ecological community to the point where it becomes unrecognisable, especially in marshes and beaches associated with estuaries, lagoons, and delta areas, such as the Albufera Natural Park [89]. It is noteworthy that *P. australis* behaves similarly to *S. patens* on the US coast, where *S. patens* is native [90]. Additionally, the changes in soil conditions caused by high salinity and drought might also affect the “unseen communities”, that is, the microorganisms that establish mutualistic relationships with plants, such as mycorrhizal fungi. Mycorrhizal communities play a vital role in maintaining plant health and many wetland ecosystem services [60]. Assessing the presence of mycorrhizae in the three host species adds extra value to the ecosystem and strengthens the need for increased investment in research in wild plant–microbe interactions. Similarly, more research on the effects of soil salinisation or drought on these microorganisms and their interaction with plants in unique ecosystems, such as the Albufera Natural Park, is needed to better understand the consequences of climate change and to develop appropriate conservation strategies.

## 4. Materials and Methods

### 4.1. Plant Material, Location, and Cultivation

**Figure 10:**
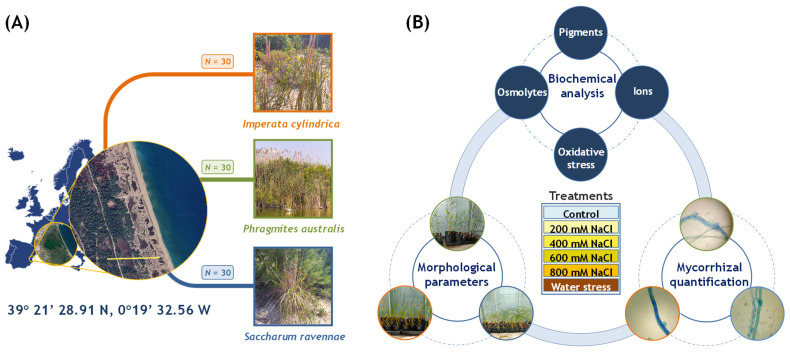
Plant sampling, treatments, and analysis. (**A**) Thirty plants from each species were randomly collected from different locations in the park. The scale corresponds to 500 m. (**B**) Plants were subjected to salt and water stress treatments after the acclimation period: 200 mM, 400 mM, 600 mM, and 800 mM NaCl, and complete withholding of irrigation (N = 5 plants per species and treatment). Control plants were watered twice weekly with 2 L of tap water (EC = 1.051 mS/cm). Plants were harvested as soon as they showed wilting symptoms. Wilt symptoms occurred after 14 days in *I. cylindrica*, 11 days in *P. australis*, and 7 days in *S. ravennae*. Then, plant morphological parameters, root mycorrhization, photosynthetic pigments, concentration of ions, osmolytes, and different aspects of the response against oxidative stress were analysed.

Plant material was provided by the Technical Management Office of the Albufera Natural Park, located in the Devesa de El Saler (Valencia Province, Spain; 39°21′28.91 N, 0°19′32.56 W). Thirty rhizomes of each selected species, *Imperata cylindrica*, *Phragmites australis*, and *Saccharum ravennae*, were collected from different locations in the park (Figure 10A). Plants had similar sizes and characteristics, but there was no age control because we wanted to investigate the effects of high salinity and water stress on the natural populations. The rhizomes were placed in individual 1 L pots and kept in a nursery within the natural park for three weeks. Then, the pots were transferred to the greenhouse at the Polytechnic University of Valencia, where they were acclimatised for two months before starting the treatments, with irrigation every five days on the lower part of the pots to simulate water uptake by the root system. The plants were kept under controlled conditions throughout the experiment, with a long day photoperiod of natural light (16 h of light and 8 h of darkness), relative humidity between 50% and 80%, and temperatures of 30 °C during the day and 20 °C at night.

After acclimation, the plants were randomly distributed in trays for each treatment. Five plant replicates were allocated to each treatment: four salt concentrations, water stress, and the control. Each tray was supplied with 2 L of salt solutions or water. The treatment was applied twice a week (on Tuesday and Friday). The plants subjected to salt treatments were irrigated with 200, 400, 600, or 800 mM NaCl solutions in tap water, respectively. Control plants were irrigated with tap water, and plants under water stress treatment were not irrigated at all. The electrical conductivity of the substrate in the pots was measured weekly with a WET sensor (Delta Devices, Cambridge, England). The treatments were applied until the plants started to show severe wilt symptoms, at which time the experiment was stopped, and morphological and biochemical analyses of each plant were carried out.

Stress conditions affected each species differently; thus, *I. cylindrica*, *P. australis*, and *S. ravennae* were harvested after 14, 11, and 7 days, respectively. Once the plants were harvested, they were transported to the laboratory to be measured and weighed in their aerial parts and roots. A weighed fraction of the aerial part was flash-frozen in liquid nitrogen and stored at −75 °C for subsequent biochemical analysis. The remaining fraction of the aerial part was reweighed (FW) and dried in an oven at 65 °C. For quantification of potential mycorrhizae structures, root fragments were cut to lengths around 0.5 cm and stored in 70% alcohol. The remaining fragments were dried in an oven at 65 °C. Dried leaves and root samples were used to measure ion contents.

### 4.2. Growth Parameters

The following parameters related to plant biomass were recorded: the increase in the number of live leaves (NL) and live stems (NT) produced during the treatment and the total fresh weight (FW) of the aerial part (FWT) and roots of each plant (FWR).

Once the aerial part and root samples reached a constant weight in the oven, they were reweighed to obtain the dry weight (DW) of the aerial part (DWT) and roots (DWR) of each plant. The water content percentage was calculated according to the following formula: WC% = [(FW − DW)/FW] × 100

### 4.3. Biochemical Analysis of Stress Markers

Different components at the molecular level potentially involved in plant responses to abiotic stress were analysed to understand the possible tolerance mechanisms activated in *I. cylindrica*, *P. australis*, and *S. ravennae*. These components included photosynthetic pigments (chlorophyll *a* and *b*, and carotenoids), three types of osmolytes (proline, total soluble sugars, and glycine betaine), ion contents (Na^+^, Cl^−^, K^+^, and Ca^2+^), biomarkers of oxidative stress (malondialdehyde and hydrogen peroxide), as well as representative antioxidant metabolites (total phenolic compounds and flavonoids) and antioxidant enzyme activities (superoxide dismutase, catalase, ascorbate peroxidase, and glutathione reductase).

### 4.4. Photosynthetic Pigments

Chlorophylls *a* and *b* and total carotenoid contents were quantified by spectrophotometric techniques according to Lichtenthaler and Wellburn [91]. Between 0.05 and 0.1 g of fresh plant material was ground and mixed with 1 mL of 80% (*v*/*v*) acetone. The samples were then shaken for 24 h in the dark. After centrifugation at 13,400× *g* for 15 min at 4 °C, the supernatant was diluted 10-fold with 80% (*v*/*v*) acetone and its absorbance was measured at 470, 646, and 663 nm. The concentration of each group of compounds was calculated according to the equations described by Lichtenthaler and Wellburn [91]. Photosynthetic pigment contents were expressed in mg g^−1^ DW.

### 4.5. Ion Contents

Concentrations of sodium (Na^+^), chloride (Cl^−^), potassium (K^+^), and calcium (Ca^2+^) were measured in leaves and roots, according to Weimberg [92]. Between 0.05 and 0.1 g of dried plant material was ground, and 2 mL of Mili-Q water was added. Samples were incubated at 95 °C in a heating block for one hour and kept shaking for 24 h in the dark. After vortexing for 5–10 s, the samples were centrifuged at 13,400× *g* for 10 min at room temperature. The supernatant was collected and diluted 15-fold. Sodium, potassium, and calcium ions were quantified using a PFP7 flame photometer (Jenway Inc., Burlington, VT, USA), whereas chlorides were measured on a MKII 926 chloride analyser (Sherwood, Inc., Cambridge, UK).

### 4.6. Osmolytes

Proline (Pro) content was quantified according to Bates et al. [93], as adapted by Vicente et al. [94]. Extracts were prepared by grinding 0.05–0.1 g of fresh leaf material in 1 mL of 3% (*w*/*v*) aqueous sulphosalicylic acid. The extracts were then centrifuged at 16,100× *g* for 15 min at 25 °C, and the supernatant was collected and mixed with acid ninhydrin and glacial acetic acid. Samples were incubated in a 98 °C water bath for 1 h, cooled on ice for 10 min, and extracted with toluene. The absorbance of the organic phase was measured at 520 nm. Proline content was expressed in μmol g^−1^ DW based on a calibration curve obtained from known proline concentrations.

Total soluble sugars (TSSs) were quantified according to Dubois et al. [95]. Extracts were prepared by grinding 0.05–0.1 g of fresh leaf material with 2 mL of 80% (*v*/*v*) methanol. Samples were mixed in a shaker for 24 h and centrifuged at 15,700× *g* for 10 min. The supernatant was collected and mixed with concentrated sulphuric acid and 5% phenol. After 20 min at room temperature, the absorbance of the samples was measured at 490 nm. TSS content was expressed as glucose equivalents (mg eq. glucose g^−1^ DW) according to a calibration curve prepared with known glucose concentrations.

Glycine betaine (GB) was quantified according to Grieve and Grattan [96]. Extracts were prepared by grinding 0.05–0.1 g of fresh leaf material with 1.5 mL of distilled water and kept in agitation for 24 h. After centrifuging the samples, 400 µL of the supernatant was mixed with 400 µL of 2 N H_2_SO_4_, and 50 µL of potassium triiodide solution was added to 125 µL of the mixture. The samples were incubated for 16 h at 4 °C in the dark. After carefully removing the supernatant, the precipitate was dissolved in 1.4 mL of 1,2-dichloroethane. After 2.5 h under cold and dark conditions, the absorbance was measured at 365 nm. The glycine betaine content was expressed in µmol g^−1^ DW based on a calibration curve obtained from known glycine betaine concentrations.

### 4.7. Oxidative Stress Biomarkers and Antioxidant Compounds

Malondialdehyde (MDA) concentration was measured to assess the oxidative stress level of the samples, whereas total phenolic compounds (TPCs) and total flavonoid (TF) contents were measured as representative antioxidant metabolites. MDA, TPC, and TF were quantified in the same methanol extracts used for TSS measurements. MDA was determined according to Hodges et al. [97] with some modifications [98]. Two methanol extracts were used per plant; to the first one, 0.5% thiobarbituric acid (TBA) prepared in 20% trichloroacetic acid (TCA) was added, whereas only 20% TCA was added to the second extract. The samples were incubated at 95 °C for 15 min and then cooled on ice for five minutes to stop the reaction. The extracts were centrifuged at 13,400× *g* for 10 min at 4 °C, and the absorbance of the supernatant was measured at 440, 532, and 600 nm. Samples containing only TCA acted as blanks for the corresponding samples with TBA + TCA. The concentration of MDA was calculated according to the equations proposed by Taulavuori et al. [98]. MDA content was expressed as nmol g^−1^ DW.

Hydrogen peroxide levels were assayed following the protocol of Alexieva et al. [99]. The leaf samples (0.05 g) were homogenised in 0.5 mL of 0.1% (*w*/*v*) trichloroacetic acid (TCA). After centrifugation at 15,700× *g* for 15 min at 4 °C, 0.5 mL of the supernatant was mixed with 0.5 mL of 10 mM Tris (pH 7) and 1 mL of 1 M KI. The reaction was run for one hour in the dark at room temperature. The absorbance was measured at 390 nm. Hydrogen peroxide content was expressed as μmol H_2_O_2_ g^−1^ DW based on a calibration curve from known hydrogen peroxide concentrations.

Total phenolic compounds (TPCs) content was measured according to the protocol of Blainski et al. [100]. Methanol extracts were mixed with the Folin–Ciocalteu reagent and 15% (*w*/*v*) sodium carbonate. Subsequently, the samples were incubated at room temperature under dark conditions for 90 min, and the absorbance was measured at 765 nm. In parallel, a calibration curve was obtained with known concentrations of gallic acid (GA), which was used as the standard. The TPC content was expressed as GA equivalents (mg eq. GA g^−1^ DW).

Total flavonoid (TF) content was quantified according to Zhishen et al. [101]. Methanol extracts were mixed with 5% (*w*/*v*) sodium nitrite, followed by 10% (*w*/*v*) aluminium chloride and 1 M sodium hydroxide. After the reaction, the absorbance was measured at 510 nm. The TF content was expressed as catechin (C) equivalents (mg eq. C g^−1^ DW) based on a calibration curve obtained with known catechin concentrations.

### 4.8. Antioxidant Enzyme Activities

Crude protein extracts were prepared according to Gil et al. [102], and proteins were quantified according to Bradford [103] using the commercial BioRad reagent and bovine serum albumin (BSA) as standard. Superoxide dismutase (SOD) activity was measured according to Beyer and Fridovich [104], considering one activity unit as the amount of enzyme causing a 50% inhibition of nitro blue tetrazolium (NBT) photoreduction, using riboflavin as a source of superoxide radicals, and measuring the absorbance at 560 nm. Catalase (CAT) activity was determined according to Aebi [105] by the decrease in absorbance at 240 nm due to the consumption of H_2_O_2_ added to the extracts. Ascorbate peroxidase (APX) activity was determined according to Nakano and Asada [106] by the decrease in absorbance at 290 nm due to ascorbate oxidation. Glutathione reductase (GR) activity was determined according to Connell and Mullet [107] by the decrease in absorbance at 340 nm due to the oxidation of the NADPH cofactor. For the three latter enzymes, one unit of enzyme activity was defined as the amount of enzyme required to oxidise one nmol of substrate per minute at 25 °C. The enzymes’ specific activities were expressed as units per gram of protein. All spectrophotometric measurements were performed on a UV-3100PC spectrophotometer (VWR International, LLC., Ragnor, PA, USA). 

### 4.9. Root Staining and Quantification of Mycorrhiza

Root samples were preserved in 70% alcohol before acidified blue Shaeffer ink staining [108]. Root fragments were washed with distilled water and cut into 0.5 cm long fragments with a scalpel. Then, the samples were incubated with 10% potassium hydroxide for 40 min in a water bath at 100 °C and washed with distilled water three times. Further, 10% Sheaffer blue dye was added until covering the roots [108], and the samples were re-incubated at 100 °C for 3 min. To remove excess dye from the roots, first, they were rinsed with 1% acetic acid and then left overnight in fresh 1% acetic acid. The next day, the acetic acid was replaced with 50% glycerol, prepared on slides with a drop of 50% lactoglycerol, and evaluated under the microscope. Arbuscules, vesicles, and hyphae were quantified according to the protocol of Trouvelot et al. [109]. Three to seven stained root fragments were evaluated under the microscope and classified according to the range of classes indicated in Figure 5. The abundance of arbuscules (% A), vesicles (% V), and hyphae (% H) in the root system was assessed (mycorrhiza manual at https://www2.dijon.inrae.fr/mychintec/Protocole/protoframe.html, accessed on 13 February 2024).

### 4.10. Statistical Analysis

Data were analysed using IBM SPSS Statistics for Windows, version 25 (IBM Corp., Armonk, NY, USA). Since some variables did not meet the variance analysis assumptions, the data were analysed using the non-parametric Kruskal–Wallis test. Pairwise post hoc comparisons were adjusted after Bonferroni. We used the ClustVis webtool [110] for a PCA analysis and the visualisation of the first three PCA components. Because the variables had different intensity ranges and units, unit variance scaling was applied as the variance normalisation method during the pre-processing. Then, we applied the NIPALSs (Nonlinear Iterative Partial Least Squares) PCA method. In addition, a clustered heatmap was produced with the three species under the six treatments with Manhattan distance and complete linkage methods. Finally, we conducted a Spearman’s rho correlation for each species and used Bonferroni correction on significant correlations. Spearman’s rho correlations were calculated with PAST 4.09 [111].

## 5. Conclusions

This study addressed the impact of high salinity and water stress on three wild plant species from the Albufera Natural Park on the Spanish Mediterranean coast, identifying *Saccharum ravennae* as the most sensitive and *Imperata cylindrica* as the most resilient to salt stress. The salt tolerance of the latter species is primarily based on an efficient mechanism of inhibition of Na^+^ and Cl^−^ transport from the roots to the aerial part of the plants (a mechanism shared by *Phragmites australis*) and the salt-induced accumulation of proline and soluble sugars in the photosynthetic tissues; K^+^ retention in the leaves in the presence of increasing Na^+^ concentrations also seemed to contribute to salt tolerance. On the other hand, the three species showed relatively high resistance to water deficit stress. All three species had roots infected with mycorrhizae, regardless of their niche of origin within the park, highlighting the importance of protecting microbial soil diversity, alongside plant and animal diversity, under the current climate change scenario.

## Figures and Tables

**Table 1:** Substrate electrical conductivity (EC). The electrical conductivity was measured weekly using a WET sensor (Delta Devices, Cambridge, England), with results reported in milli Siemens per centimetre (mS/cm). The plants were monitored until they exhibited wilting symptoms. Wilting occurred after 14 days for *Imperata cylindrica*, 11 days for *Phragmites australis*, and 7 days for *Saccharum ravennae*. Different lowercase letters indicate significant differences between treatments for each day and species, and different uppercase letters indicate significant differences across different days for each treatment and species.

Species	*I. cylindrica*			*P. australis*			*S. ravennae*	
Day	0	7	14	0	7	11	0	7
Treatment								
Control	2.0 ± 0.2 ^A^	1.3 ± 0.2 ^bA^	1.8 ± 0.4 ^bcA^	2.0 ± 0.1 ^abB^	1.1 ± 0.1 ^abA^	1.3 ± 0.1 ^bcA^	1.5 ± 0.2 ^bA^	1.1 ± 0.1 ^cB^
0.2 M NaCl	1.8 ± 0.1 ^B^	2.7 ± 0.1 ^abB^	7.0 ± 0.3 ^abcA^	2.0 ± 0.1 ^abA^	3.5 ± 0.6 ^abA^	5.3 ± 1.4 ^abcA^	1.4 ± 0.2 ^bB^	3.8 ± 0.3 ^abcA^
0.4 M NaCl	1.8 ± 0.1 ^B^	3.8 ± 0.5 ^abB^	30.3 ± 5.2 ^abA^	2.3 ± 0.1 ^aB^	5.4 ± 0.8 ^abAB^	9.9 ± 2.4 ^abcA^	2.3 ± 0.2 ^abB^	9.1 ± 1.2 ^abcA^
0.6 M NaCl	2.3 ± 0.1 ^B^	4.1 ± 3.0 ^abB^	40.2 ± 14.8 ^abA^	2.4 ± 0.1 ^aB^	7.7 ± 2.6 ^aAB^	42.4 ± 16.2 ^abA^	2.3 ± 0.1 ^abB^	18.7 ± 4.5 ^abA^
0.8 M NaCl	2.2 ± 0.1 ^B^	8.8 ± 1.4 ^aB^	53.8 ± 0.0 ^aA^	2.2 ± 0.1 ^abB^	20.8 ± 14.3 ^aAB^	46.7 ± 0.0 ^aA^	2 ± 0.1 ^abB^	46.9 ± 6.9 ^aA^

**Table 2:** Kruskal–Wallis analysis for overall main treatment effects on plant biomass, biochemical response, and mycorrhizal colonisation. Thirty plants of each species (five plants per treatment) were subjected to four salt concentrations (200, 400, 600, and 800 mM NaCl) and water stress (withholding of irrigation) on pots containing fresh soil from the Albufera Natural Park. Control plants were watered twice weekly with 2 L of tap water (EC = 1.051 mS/cm). Treatment lengths were 14 days for *I. cylindrica*, 11 days for *P. australis*, and seven days for *S. ravennae*. Values in bold denote statistically significant differences.

Species	*I. cylindrica*			*P. australis*			*S. ravennae*		
Variable	*N*	*χ*2	*p*-Value	*N*	*χ*2	*p*-Value	*N*	*χ*2	*p*-Value
**BIOMASS**									
Leaf fresh weight	30	21.263	**0.001**	26	4.055	0.542	28	21.012	**0.001**
Root fresh weight	30	11.147	**0.049**	27	8.230	0.144	30	8.055	0.153
Leaves produced	30	23.810	**<0.001**	25	15.749	**0.008**	30	23.882	**<0.001**
Stems produced	30	13.340	**0.020**	29	16.393	**0.006**	30	12.425	**0.029**
Leaf water content	26	8.809	0.117	27	9.756	0.082	29	16.531	**0.005**
Root water content	30	8.310	0.140	27	6.140	0.293	29	7.853	0.165
Root/shoot ratio	26	19.464	**0.002**	26	7.007	0.220	26	11.073	**0.050**
**PIGMENTS**									
Chlorophyll *a*	26	15.166	**0.010**	19	4.947	0.422	26	14.850	**0.011**
Chlorophyll *b*	26	11.669	**0.040**	19	4.211	0.519	26	14.786	**0.011**
Carotenoids	26	15.329	**0.009**	18	6.763	0.239	27	10.552	0.061
Chlorophylls/carotenoids	25	14.720	**0.012**	18	2.986	0.702	27	6.943	0.225
**IONS**									
Na^+^ in roots	30	25.377	**<0.001**	27	19.976	**0.001**	29	24.061	**<0.001**
Na^+^ in leaves	28	22.154	**<0.001**	25	3.574	0.612	23	8.441	0.134
Cl^−^ in roots	30	25.036	**<0.001**	27	19.084	**0.002**	30	20.102	**0.001**
Cl^−^ in leaves	28	19.482	**0.002**	27	12.680	**0.027**	24	17.460	**0.004**
K^+^ in roots	30	11.921	**0.036**	26	7.805	0.167	29	12.723	**0.026**
K^+^ in leaves	29	8.685	0.122	27	4.814	0.439	24	7.098	0.213
Ca^2+^ in roots	30	24.272	**<0.001**	27	17.121	**0.004**	30	20.055	**0.001**
Ca^2+^ in leaves	29	23.600	**<0.001**	27	7.042	0.218	24	18.885	**0.002**
**OSMOLYTES**									
Proline	26	15.440	**0.009**	20	6.972	0.223	25	12.883	**0.024**
Total soluble sugars	24	14.186	**0.014**	17	1.686	0.891	25	7.751	0.170
Glycine betaine	24	3.477	0.627	21	2.288	0.808	27	5.362	0.373
**OXIDATIVE STRESS MARKERS**									
Malondialdehyde	23	11.982	**0.035**	19	2.411	0.790	26	9.390	0.094
Hydrogen peroxide	24	3.802	0.578	18	3.973	0.553	27	7.410	0.192
**NON-ENZYMATIC ANTIOXIDANTS**									
Total phenolic compounds	25	11.346	**0.045**	19	0.180	0.999	26	7.716	0.173
Total flavonoids	25	11.527	**0.042**	19	0.816	0.976	25	6.295	0.279
**TOTAL SOLUBLE PROTEINS**									
Total soluble proteins	23	4.145	0.529	19	4.589	0.468	28	18.015	**0.003**
**ANTIOXIDANT ENZYMES**									
Superoxide dismutase	22	4.040	0.544	19	4.054	0.542	24	8.798	0.117
Catalase	22	3.519	0.621	21	5.747	0.332	28	3.596	0.609
Ascorbate peroxidase	23	10.954	0.052	20	2.654	0.753	26	3.817	0.576
Glutathione reductase	23	5.892	0.317	21	2.152	0.828	27	11.836	**0.037**
**MYCORRHIZAE ANALYSIS**									
Arbuscule abundance	24	4.012	0.548	13	3.899	0.564	26	3.133	0.679
Vesicle abundance	23	4.769	0.445	13	8.189	0.146	26	4.637	0.462
Hyphae abundance	22	3.961	0.555	13	4.292	0.508	26	2.526	0.773

**Table 3:** Summary of oxidative stress markers, antioxidant compounds, soluble proteins, and antioxidant enzyme activities from *Imperata cylindrica*, *Phragmites australis*, and *Saccharum ravennae*. Thirty plants of each species (five plants per treatment) were exposed to 200, 400, 600, and 800 mM NaCl and water stress (WS) on pots containing fresh soil from the Albufera Natural Park. Control plants were watered twice weekly with 2 L of tap water (EC = 1.051 mS/cm). Treatment lengths were 14 days for *I. cylindrica*, 11 days for *P. australis*, and 7 days for *S. ravennae*. Letters represent post hoc pairwise statistically significant differences corrected after Bonferroni between treatments. Mean ± SE. values are shown. Abbreviations: dry weight (DW), catechin (C), gallic acid (GA), superoxide dismutase (SOD), catalase (CAT), ascorbate peroxidase (APX), and glutathione reductase (GR).

Parameter	Treatment	*N*	*I. cylindrica*	*N*	*P. australis*	*N*	*S. ravennae*
Malondialdehyde (nmol g^−1^ DW)	Control	5	17.6 ± 9.0 ^a^	4	96.2 ± 12.3 ^a^	4	31.9 ± 11.8 ^a^
	200 mM	5	17.4 ± 9.8 ^a^	5	78.7 ± 18.5 ^a^	4	0.0 ± 0.0 ^a^
	400 mM	5	0.0 ± 0.0 ^a^	3	78.3 ± 23.1 ^a^	5	35.9 ± 21.0 ^a^
	600 mM	3	0.0 ± 0.0 ^a^	2	94.8 ± 13.8 ^a^	5	41.5 ± 27.1 ^a^
	800 mM	1	6.3 ± 0.0 ^a^	2	65.3 ± 5.5 ^a^	4	0.7 ± 0.7 ^a^
	WS	4	31.7 ± 11.0 ^a^	3	95.5 ± 30.2 ^a^	4	0.0 ± 0.0 ^a^
Hydrogen peroxide (µmol H_2_O_2_ g^−1^ DW)	Control	5	4.6 ± 0.5 ^a^	3	0.6 ± 0.1 ^a^	5	5.0 ± 1.4 ^a^
	200 mM	5	3.8 ± 0.5 ^a^	5	0.7 ± 0.1 ^a^	4	6.0 ± 1.4 ^a^
	400 mM	5	5.4 ± 0.8 ^a^	2	0.3 ± 0.2 ^a^	5	2.2 ± 0.7 ^a^
	600 mM	4	3.7 ± 1.0 ^a^	2	0.4 ± 0.4 ^a^	5	2.9 ± 0.5 ^a^
	800 mM	1	3.3 ± 0.0 ^a^	2	0.2 ± 0.2 ^a^	4	4.0 ± 2.1 ^a^
	WS	4	4.7 ± 0.5 ^a^	4	0.4 ± 0.2 ^a^	4	4.2 ± 0.5 ^a^
Total phenolic compounds (mg eq. GA g^−1^ DW)	Control	5	2.2 ± 0.3 ^a^	4	6.6 ± 2.3 ^a^	4	2.2 ± 0.1 ^a^
	200 mM	5	2.1 ± 0.2 ^a^	5	4.5 ± 1.3 ^a^	4	2.8 ± 0.5 ^a^
	400 mM	5	2.7 ± 0.4 ^a^	2	5.8 ± 2.7 ^a^	5	1.3 ± 0.4 ^a^
	600 mM	4	3.2 ± 0.3 ^a^	2	4.5 ± 0.4 ^a^	5	2.0 ± 0.3 ^a^
	800 mM	1	1.3 ± 0.0 ^a^	2	4.3 ± 0.1 ^a^	4	1.5 ± 0.6 ^a^
	WS	5	1.7 ± 0.1 ^a^	4	4.4 ± 0.3 ^a^	4	1.6 ± 0.3 ^a^
Total flavonoids (mg eq. C g^−1^ DW)	Control	5	4.3 ± 0.6 ^ab^	4	2.4 ± 0.8 ^a^	4	3.3 ± 0.3 ^a^
	200 mM	5	4.9 ± 0.7 ^ab^	4	1.7 ± 0.7 ^a^	4	5.4 ± 1.1 ^a^
	400 mM	5	5.9 ± 1.1 ^ab^	3	1.5 ± 0.5 ^a^	5	2.3 ± 0.8 ^a^
	600 mM	4	6.5 ± 0.5a	2	1.6 ± 0.3 ^a^	5	3.7 ± 0.4 ^a^
	800 mM	1	2.8 ± 0.0 ^ab^	2	1.5 ± 0.1 ^a^	3	3.0 ± 2.0 ^a^
	WS	5	3.4 ± 0.3 ^b^	4	1.7 ± 0.3 ^a^	4	2.6 ± 0.8 ^a^
Total soluble proteins (mg protein g^−1^ DW)	Control	5	2.2 ± 0.3 ^a^	4	3.71 ± 0.4 ^a^	5	2.5 ± 0.6 ^a b^
	200 mM	5	2.1 ± 0.4 ^a^	4	3.03 ± 0.3 ^a^	4	4.1 ± 0.5 ^a b^
	400 mM	4	2.7 ± 0.4 ^a^	3	2.8 ± 0.2 ^a^	5	1.4 ± 0.4 ^b^
	600 mM	4	2.7 ± 0.4 ^a^	2	3.1 ± 0.4 ^a^	5	2.5 ± 0.3 ^b^
	800 mM	1	2.0 ± 0.0 ^a^	2	2.7 ± 0.3 ^a^	4	1.3 ± 0.4 ^a b^
	WS	4	1.9 ± 0.3 ^a^	4	2.9 ± 0.3 ^a^	5	4.2 ± 0.4a
SOD activity (U g^−1^ protein)	Control	5	388.7 ± 27.1 ^a^	4	137.2 ± 49.8 ^a^	4	145.2 ± 46.4 ^a^
	200 mM	5	429.1 ± 51.1 ^a^	5	287.3 ± 105.2 ^a^	4	433.1 ± 79.0 ^a^
	400 mM	4	415.0 ± 53.2 ^a^	3	280.7 ± 151.6 ^a^	4	185.0 ± 44.6 ^a^
	600 mM	4	484.2 ± 80.2 ^a^	2	421.3 ± 213.5 ^a^	5	544.4 ± 153.4 ^a^
	800 mM	1	198.7 ± 0.0 ^a^	2	365.4 ± 4.6 ^a^	3	241.1 ± 166.1 ^a^
	WS	3	447.1 ± 57.7 ^a^	3	330.0 ± 54.0 ^a^	4	275.5 ± 103.0 ^a^
CAT activity (U g^−1^ protein)	Control	4	3.5 ± 1.3 ^a^	5	6.0 ± 4.0 ^a^	5	6.06 ± 4.4 ^a^
	200 mM	5	17.6 ± 6.7 ^a^	5	13.6 ± 7.1 ^a^	4	12.32 ± 5.5 ^a^
	400 mM	4	10.2 ± 2.4 ^a^	3	35.0 ± 33.0 ^a^	5	35.1 ± 23.4 ^a^
	600 mM	4	13.0 ± 7.0 ^a^	2	28.1 ± 8.5 ^a^	5	4.5 ± 2.7 ^a^
	800 mM	1	3.9 ± 0.0 ^a^	2	90.2 ± 72.4 ^a^	4	25.2 ± 18.2 ^a^
	WS	4	12.4 ± 6.3 ^a^	4	22.8 ± 14.3 ^a^	5	5.6 ± 3.8 ^a^
APX activity (U g^−1^ protein)	Control	5	293.5 ± 47.9 ^a^	4	1287.7 ± 264.8 ^a^	5	322.0 ± 112.9 ^a^
	200 mM	5	454.7 ± 68.4 ^a^	5	1436.4 ± 398.4 ^a^	4	526.5 ± 76.6 ^a^
	400 mM	4	391.4 ± 107.7 ^a^	3	1000.3 ± 214.9 ^a^	5	531.9 ± 167.4 ^a^
	600 mM	4	665.2 ± 142.2 ^a^	2	1660.6 ± 667.6 ^a^	4	536.6 ± 28.7 ^a^
	800 mM	1	413.6 ± 0.0 ^a^	2	1413.5 ± 112.0 ^a^	3	283.9 ± 174. 2 ^a^
	WS	4	706.4 ± 144.8 ^a^	4	1040.7 ± 369.6 ^a^	5	637.5 ± 132.9 ^a^
GR activity (U g^−1^ protein)	Control	5	27.3 ± 5.3 ^a^	5	1.2 ± 1.1 ^a^	4	13.2 ± 6.9 ^a^
	200 mM	5	22.6 ± 6.3 ^a^	5	0.2 ± 0.2 ^a^	4	15. 0 ± 6.5 ^a^
	400 mM	4	12.6 ± 7.7 ^a^	3	21.6 ± 21.1 ^a^	5	28.1 ± 9.9 ^a^
	600 mM	4	27.8 ± 13.6 ^a^	2	5.4 ± 5.4 ^a^	5	17.9 ± 9.3 ^a^
	800 mM	1	35.5 ± 0.0 ^a^	2	0.0 ± 0.0 ^a^	4	21.5 ± 5.7 ^a^
	WS	4	41.7 ± 7.8 ^a^	4	0.3 ± 0.3 ^a^	5	4.3 ± 3.5 ^a^

## Data Availability

Data are available upon request.
